# Job-search self-efficacy in unemployed individuals with mental health issues: investigating the role of self-stigma with variable-centered and person-centered approaches

**DOI:** 10.1186/s40359-026-04193-2

**Published:** 2026-03-19

**Authors:** Svenja Schlachter, Melanie Gantner, Maria Gralla, Marina Pumptow, Sophia Helen Adam, Rebecca Erschens, Harald Gündel, Jörn von Wietersheim, Nicolas Rüsch

**Affiliations:** 1 Department of Psychiatry and Psychotherapy II, Ulm University and BKH Günzburg, Parkstraße 11, Ulm, 89073 Germany; 2 Department of Psychosomatic Medicine and Psychotherapy, Ulm University Medical Center, Albert-Einstein-Allee 23, Ulm, 89081 Germany; 3 German Center for Mental Health (DZPG), partner site Mannheim-Heidelberg-Ulm/ZIHUb, Ulm, Germany; 4 Institute for Clinical Epidemiology and Applied Biometry, University Hospital Tübingen and Medical Faculty, University of Tübingen, Silcherstr. 5, Tübingen, 72076 Germany; 5 Department of Psychosomatic Medicine and Psychotherapy, University Hospital Tübingen, Osianderstraße 5, Tübingen, 72076 Germany; 6 German Centre for Mental Health (DZPG), partner site Tübingen, Tübingen, Germany

**Keywords:** Self-stigma, Mental health, “Why try” effect, Unemployment, Job-search self-efficacy, Job-search behavior, Vocational rehabilitation

## Abstract

**Background:**

Unemployed individuals with mental health issues frequently struggle to find re-employment. The relevance of self-stigma, that is, applying public stigma toward mental health issues to oneself, has been under-researched in this context, but might provide valuable insights into why finding re-employment is particularly challenging for these individuals. Drawing on the “why try” effect of self-stigma and the conservation of resources theory, we examined the associations between self-stigma, job-search self-efficacy, and job-search behavior, also considering the role of social inclusion, readiness to find employment, psychological distress, and having a diagnosed mental health condition.

**Methods:**

As part of a wider intervention project, we collected baseline data via standardized questionnaires and structured interviews and applied both variable- and person-centered analysis approaches. Data were provided by 422 unemployed individuals with mental health issues in Germany.

**Results:**

Variable-centered analysis indicated a negative indirect effect of self-stigma on job-search behavior through reduced job-search self-efficacy, supporting a domain-specific “why try” effect. This indirect effect was not conditional on other variables. Readiness to find employment, however, moderated the link between self-stigma and job-search self-efficacy with higher readiness exacerbating the negative association. Person-centered analysis identified four distinct profiles of unemployed individuals with mental health issues: Whereas two profiles showed mostly quantitative differences in the magnitude of the variable scores (i.e., low vs. high levels of self-stigma, psychological distress, and social inclusion), two profiles showed qualitatively distinct combinations of self-stigma and psychological distress. The four profiles also differed regarding job-search self-efficacy levels with more favorable profile compositions showing higher levels as opposed to less favorable profile compositions.

**Conclusions:**

Our findings support the relevance of self-stigma for job-search self-efficacy and, indirectly, job search. Implications for research and practice, particularly in terms of possible interventions regarding self-stigma in the context of vocational rehabilitation, are discussed.

**Trial registration:**

The wider intervention project has been registered with the German Clinical Trials Register: DRKS00029002 (registered on 11 May 2022).

## Background

Unemployment is a major concern from both economic and public health perspectives. Economically, it imposes substantial direct costs on welfare states through increased government expenditure on unemployment benefits as well as lost revenue from social insurance contributions and income tax [1–3]. It also entails indirect costs due to reduced work-related economic productivity. From a public health perspective, unemployment is closely and reciprocally linked to mental health issues: Unemployment is associated with poor mental health [4–6], while poor mental health, in turn, is associated with increased probability of becoming [4, 6] and remaining unemployed [7, 8]. Re-employment, conversely, is associated with improvement in mental health [6, 9]. Given its substantial economic burden and its strong, bidirectional association with mental health, reducing unemployment constitutes a key target for both economic policy and public mental health interventions. Understanding and addressing the barriers to re-employment among individuals with mental health issues represents a critical point in this context.

The reasons why individuals with mental health issues struggle to find and maintain re-employment are manifold and complex. One contributing factor is that mental health issues and unemployment are both highly adverse experiences, each depleting resources and coping capacity, even more so when combined [10, 11]. Accordingly, a person’s capacity to work and confidence in their work-related capabilities can be negatively affected by mental health issues, particularly during episodes of high symptom burden [12, 13] and after recent hospitalization due to a mental health condition [14]. As a result, mental health issues and unemployment are commonly accompanied by negative emotions and cognitions about oneself, such as low self-worth and self-efficacy [11]. In addition, unemployed individuals with mental health issues frequently encounter public stigma toward them, for either their unemployment [11, 15] or mental health issues [16, 17], or both [18]. Such stigmas can negatively affect employers’ decisions regarding hiring and continued employment [19, 20]. Consequentially, individuals’ capacity to engage in successful job search and self-presentation to potential employers is impeded. A lack of commitment to employment, on the other hand, does not appear to be a predominant impediment to re-employment, as many unemployed individuals, including those with mental health issues, have a strong desire to work [21, 22].

A further contributing factor in this context could be self-stigma. Self-stigma occurs when affected individuals apply public stigma toward mental health issues to themselves [23]. This process undermines their self-worth and self-efficacy [24–26], and can result in demoralization and hopelessness, and consequentially in avoidant coping strategies and withdrawal from goal-oriented efforts aimed at improving one’s situation [26]—a phenomenon termed the *“why try” effect* [27]. The relevance of self-stigma in terms of vocational functioning, particularly regarding prolonged unemployment and job-search efforts, has been proposed [19], but has received less research attention than the more direct emotional outcomes of self-stigma [28].

To advance our understanding of the factors contributing to prolonged unemployment in the context of mental health issues, we examine several predictors of job-search self-efficacy and job-search behavior, focusing on self-stigma and using both variable-centered and person-centered approaches. Taking a variable-centered approach, we examine the process of how individuals’ self-stigma might affect their job-search behavior through reduced self-efficacy to find employment. Given that meta-analyses found heterogenous effects between mental health issues and finding re-employment [6], we assume that some individuals struggle more than others in this context. Consequentially, we examine how the process between self-stigma and job-search behavior might differ based on individuals’ stressors and resources. More specifically, we examine social inclusion and readiness to find employment as mitigating resources, and psychological distress and having a diagnosed mental health condition as exacerbating stressors. To inform our research model, we draw on the “why try” effect of self-stigma [27] to explain the process, as well as the *conservation of resources* (COR) *theory* [29] to propose boundary conditions. Additionally, using a person-centered approach, we explore whether certain profiles of unemployed individuals can be identified based on self-stigma and the proposed boundary conditions, and how these profiles, in turn, relate to job-search self-efficacy. To test our propositions, we used data from a sample of 422 unemployed individuals with mental health issues recruited in job centers in Southern Germany.

We aim to make several contributions to the research area of unemployment and mental health. As the associations between poor mental health and unemployment are manifold, various leverage points have to be identified to support unemployed individuals with mental health issues in finding and maintaining re-employment. Self-stigma could be such a leverage point which is an under-researched area. Additionally, unemployed individuals are a heterogenous sample [30, 31] and potential predictors, especially those related to mental health, might be highly interrelated. A person-centered approach which identifies different configurations of subgroups of this heterogenous sample and how these configurations relate to job-search self-efficacy would advance our understanding of how various factors in combination could contribute to continued unemployment. Overall, by taking two different analysis approaches, we aim to obtain complementary perspectives on why many unemployed individuals with mental health issues feel unconfident in finding employment, thus not attempting it [32], providing insights for vocational rehabilitation interventions.

## Theoretical background

### Variable-centered perspective on self-stigma, job-search self-efficacy, and job-search behavior

In vocational research, variable-centered approaches are commonly used and examine research questions regarding associations between variables across individuals. These approaches are more parsimonious and easily interpretable in terms of tendencies in populations in contrast to person-centered approaches [32].

#### Self-stigma and job-search self-efficacy

Self-stigma is a progressive process when a person is aware of negative stereotypes about individuals with mental health conditions, agrees with them and, ultimately, applies these stereotypes to themselves, thus feeling prejudiced toward themselves [23, 33]. In other words, self-stigma is “the way in which people with mental health conditions see themselves as being mentally unwell and, therefore, of lesser value” (p. 1442) [34]. It is important to stress that self-stigma is a consequence of public stigma and not the individuals’ fault who experience it [16, 34]. Self-stigma is, however, not experienced by everyone with mental health issues to the same extent: Whereas some individuals internalize public stigma to a higher extent, others react to public stigma with righteous anger, yet others are indifferent to public stigma toward them [23, 25].

Internalized negative beliefs of oneself due to self-stigma result in negative emotional and cognitive reactions such as reduced self-worth and self-efficacy [23, 25, 28]. Self-efficacy, in turn, is associated with behavioral persistence despite difficulties and setbacks [35]. In this study, we focus on a domain-specific type of self-efficacy, namely an unemployed person’s self-efficacy in terms of the job-search process, that is, job-search self-efficacy. It represents a positive self-evaluation regarding one’s confidence in having the relevant skills to perform job-search relevant activities, thus ultimately obtaining employment [36, 37]. We hypothesize the following:

- Hypothesis 1: Self-stigma is negatively associated with job-search self-efficacy.

#### The “why try” effect of self-stigma on job-search behavior

Self-stigma has not only been associated with negative emotional and cognitive reactions, but also behavioral consequences in terms of poorer psychosocial functioning, including functioning related to employment [25, 38]. In an early study on such consequences, Link [39] found that individuals who have been given an official diagnosis are less likely to find and maintain employment. He argued that part of this effect can be attributed to individuals having internalized negative beliefs and thus feel less confident in their abilities in terms of employment. Similarly, Yanos et al. [26, 28] proposed and empirically found that self-stigmatizing individuals experience heightened hopelessness, resulting in resignation and avoidance of active engagement in rehabilitation attempts, which in turn harms their vocational functioning. Consistent with these propositions, Waynor et al. [14] found that individuals who experience self-stigma also report lower work-related self-efficacy, reflecting reduced confidence in their ability to engage effectively in work-related tasks. When self-stigmatizing individuals perceive their work-related abilities as limited, it is unlikely that they perceive re-employment efforts as worthwhile.

The process regarding the negative effect of self-stigma on psychosocial functioning is explained by the “why try” effect [27]. It states that individuals who experience self-stigma suffer from loss of self-worth and self-efficacy, which results in their demoralization [40]. Subsequently, individuals give up trying to engage in goal-directed behaviors in important life domains as they expect their efforts to be futile. This disengagement from efforts to improve one’s situation is considered a behavioral consequence of self-stigma [27]. Goal-directed behaviors include life domains such as independent living, social engagement, seeking treatment for mental health conditions, finding employment, or engaging in vocational rehabilitation programs.

Job-search behavior is a problem-focused coping strategy when being unemployed [5, 41]. It is associated with positive job-search outcomes, such as more job offers, higher chances of re-employment and shorter unemployment duration [42, 43]. Despite being an effective behavior to gain re-employment, it is an effortful and frequently discouraging process with experiences of rejections, uncertainty, and fatigue [11, 44, 45]. Job-search behavior is therefore considered a self-regulatory process, requiring self-regulatory resources to implement and maintain it [42, 43, 46], with job-search self-efficacy being such a resource [46, 47]. Accordingly, being confident in finding employment has been found to be associated with higher levels of job-search behavior [42, 43, 46] and a higher conversion rate between job interviews into actual offers [44].

Consequently, applying the “why try” effect to unemployed individuals with mental health issues, we argue that these individuals feel helpless and discouraged regarding their ability to find employment if they report high levels of self-stigma. Consequently, they stop trying, considering their job-search activities to be futile. This aligns with research related to job-search behavior arguing that perceiving situational control is an important predictor of active job search [41, 48]. Accordingly, we propose that self-stigma in unemployed individuals reduces engagement in job-search behaviors by reducing job-search self-efficacy. Our hypothesis is the following:

- Hypothesis 2: There is a negative indirect effect between self-stigma and job-search behavior, mediated by job-search self-efficacy.

#### Boundary conditions of the negative association between self-stigma and job-search behavior

In the next step, we examine whether there are boundary conditions to the proposed negative association between self-stigma and job-search self-efficacy, also affecting the indirect effect between self-stigma and job-search behavior. Drawing on COR theory, we argue that certain resources in the context of unemployment and mental health can mitigate the negative association, whereas further stressors might exacerbate it. COR theory is a motivational theory which states that individuals aim to accumulate and maintain resources to support goal achievement. If resources are threatened, lost, or not sufficiently gained despite resource investment, individuals experience stress [29]. To offset resource loss and cope with experienced stress, individuals can engage in coping strategies aimed at conserving existing resources and compensating for losses through resource substitution or replacement [29, 49]. If resources become increasingly depleted, individuals struggle to replenish them, resulting in a downward loss spiral where resource loss begets further resource loss [49].

Unemployment and mental health issues are both highly stressful as they threaten and drain individuals’ resources [4, 45]. In terms of unemployment, individuals lose various resources provided by employment such as a stable income, time structure, activation in daily life, social contact, identity and status as well as contributing to a collective purpose [11, 50, 51]. Mental health issues also threaten and drain resources through their frequently considerable symptom burden. Additionally, they are commonly stigmatized in society, resulting in discrimination, including being denied access to resources in various areas of life [16, 34, 52]. Experiencing self-stigma is another stressor in this context, draining resources further [53]. We therefore conclude that many unemployed individuals with mental health issues, particularly those that experience self-stigma, have a limited supply of resources, making them less capable to cope with stress and thus prone to further resource loss, meaning they are caught in loss spirals which are difficult to break [10]. We propose that resources which some individuals have at their disposal can substitute lost resources and therefore help to break or mitigate these loss spirals, thus improving individuals’ mental health and self-efficacy [5]. Consequently, they have more resources to invest into effortful job-search behavior. In contrast, others experience further stressors, taxing their resource pool even more, which accelerates their loss spiral.

##### Social inclusion

Social inclusion—defined as experiencing agency and participation in societal activities, feeling a sense of belonging, and being socially connected to one’s community [54, 55]—is crucial for mental health [56]. Mental health issues, in turn, frequently result in social exclusion [16, 52]. An important part of social inclusion is employment as it provides social contacts, status, and contributing to a shared purpose [50]. Losing employment, individuals also lose this part of belonging and agency, resulting in lower levels of social inclusion and mental health [51, 55].

Social inclusion and support are key resources for coping with the stress of unemployment, both in terms of mitigating negative consequences for mental health [5], as well as maintaining or encouraging job-search self-efficacy and behavior [42, 57, 58]. In contrast, social exclusion following job loss has been linked to increased fatigue, which negatively affects re-employment quality [45]. Although unemployment depletes resources, individuals differ in how strongly their social resources and perception of social inclusion are affected by it [45, 55], with some retaining or accessing alternative social resources that can substitute lost resources. We therefore propose that higher perceived social inclusion during unemployment and mental health issues buffers the negative impact of self-stigma on job-search behavior by weakening its association with job-search self-efficacy. Our hypothesis is the following:

- Hypothesis 3: Social inclusion moderates the negative indirect effect between self-stigma and job-search behavior, mediated by job-search self-efficacy, such that the negative indirect effect is weakened when social inclusion is high.

##### Readiness to find employment

The concept of readiness to find employment is operationalized based on the individual’s stage in the process of intentional change toward finding employment. The stages of intentional change are based on the *transtheoretical model of change* which classifies a person’s process toward a behavioral change into five stages: precontemplation, contemplation, preparation, action, and maintenance [59, 60]. In the initial stages, behavioral intentions for change are gradually formed, progressing in later stages to enacting the behavioral change. The higher the stage, the more likely successful behavioral change becomes [61].

Finding employment requires effort and self-regulation [42]. Strong motives to find employment, especially autonomous job-search motivation, have been argued to be relevant for self-regulatory goal-setting and strategy development regarding job search [62], as well as for engaging in and maintaining job-search efforts despite frustrations [42, 63]. A more advanced stage of behavioral change is associated with stronger motivation, commitment, and capacity to manage behavioral change [60]. Consequently, it can be considered a personal resource in the context of COR theory, thus a probable buffer of the negative, resource-draining effect of self-stigma. We therefore propose that being on a more advanced stage of behavioral change toward finding re-employment functions as a resource mitigating the negative association between self-stigma and job-search self-efficacy, and therefore the indirect effect between self-stigma and job-search behavior. We accordingly propose the following hypothesis:

- Hypothesis 4: Readiness to find employment moderates the negative indirect effect between self-stigma and job-search behavior, mediated by job-search self-efficacy, such that the negative indirect effect is weakened for individuals in a higher stage of readiness.

##### Psychological distress

Stressful experiences can result in feeling psychological distress, an indicator of poor mental health characterized by a set of aversive mental and physical symptoms [64]. Experiencing psychological distress is a stressor which drains a person’s resource pool, thus accelerating loss spirals.

Unemployment is a stressful event which depletes individuals’ resources and affects mental health. Meta-analyses have shown, however, that these associations can vary between individuals [5, 6]: Some might experience more stress due to other stressors in their environment, whereas others have more resources to draw on to buffer the stressful experience. Experiencing higher levels of non-specific psychological distress, that is, distress not only due to unemployment, but also other stressful events, can affect individuals’ perception of self-efficacy [65] and leaves only limited resources to engage in effortful job-search behavior. Consequently, we propose that high levels of psychological distress will exacerbate the indirect effect between self-stigma and job-search behavior by strengthening the negative association between self-stigma and job-search self-efficacy. The hypothesis is the following:

- Hypothesis 5: Psychological distress moderates the negative indirect effect between self-stigma and job-search behavior, mediated by job-search self-efficacy, such that the negative indirect effect is exacerbated when psychological distress is high.

##### Diagnosis of mental health condition

According to labeling theory, a formal diagnosis of a mental health condition can become a source of psychosocial stress beyond the burden of symptoms themselves [39, 66]. Through socialization, the label of a mental health condition carries strong beliefs about affected individuals; beliefs which are activated when receiving this label and applying them to oneself [66, 67]. As a result, being formally labelled as mentally unwell can induce psychosocial vulnerability [66], undermining self-confidence and leading individuals to avoid situations where they fear rejection [39, 66].

In terms of employment, having a diagnosed mental health condition has been found to be negatively associated with employment status, above and beyond functional impairment due to the mental health condition itself [39, 67]. We propose that having a diagnosis operates as an additional stressor that depletes individual resources, thereby reducing the capacity to invest in resource-intensive job-search behavior. Our hypothesis is the following:

- Hypothesis 6: Having a diagnosis of a mental health condition moderates the negative indirect effect between self-stigma and job-search behavior, mediated by job-search self-efficacy, such that the negative indirect effect is exacerbated when there is a diagnosis.

**Fig. 1:**
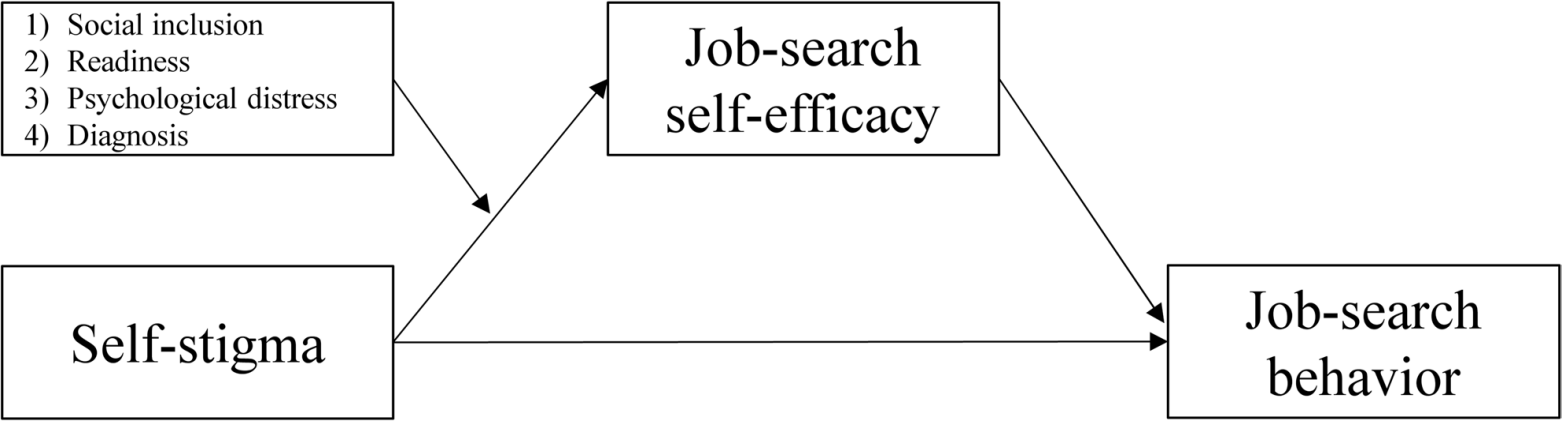
Research model for the variable-centered approach

The research model for the variable-centered approach is illustrated in Fig. 1.

### Person-centered perspective on unemployed individuals with mental health issues and job-search self-efficacy

Person-centered approaches are less commonly used in vocational research, but growing in popularity [68, 69]. These approaches focus on identifying configurations of variables in populations, thus categorizing individuals into subpopulations, also examining how subpopulation membership is associated with predictor or outcome variables. In contrast to variable-centered approaches, these approaches loosen the idea of homogeneity in populations [68, 70]. Additionally, person-centered approaches allow researchers to examine the interplay of various variables beyond what is practically feasible or interpretable with multi-way interactions in a variable-centered approach [68].

In the present study, we aim to advance our understanding of contributing factors to lacking job-search self-efficacy in a sample of unemployed individuals with mental health issues. As the proposed contributing factors are likely to be interrelated (e.g., self-stigma and social inclusion), taking a person-centered approach enables us to understand varying compositions of variables which might affect job-search self-efficacy. Accordingly, we consider the person-centered approach a complementary perspective to studying the present population of unemployed individuals with mental health issues [32, 70, 71]. Given the inductive nature of the person-centered approach, we do not propose formal hypotheses regarding the emerging profiles but rather pose research questions relating to identification of distinct profiles in our sample and how these profiles relate to differing levels of job-search self-efficacy. Our research questions are the following:

*Research Question 1: Are there quantitatively and qualitatively distinct profiles of unemployed individuals with mental health issues?*

*Research Question 2: Do profiles of unemployed individuals with mental health issues relate to job-search self-efficacy?*

## Methods

The present data uses baseline data from a wider intervention project. The wider intervention project is further described in Schlachter et al. [72].

### Participants and procedure

We recruited participants in collaboration with six job centers in Southern Germany. Job centers in Germany are public agencies which provide basic income support and employment services for unemployed individuals. Initially, individuals had to fulfill five general criteria, namely (1) being a formally registered client at the job center, (2) having been registered in that job center for full basic income support based on Germany’s Second Book of the Social Code for less than six months, (3) not currently being employed, (4) being between 18 and 60 years of age, and (5) having sufficient knowledge of German. Fulfilling these criteria, they were asked to complete *Kessler’s Psychological Distress Scale* – K6 version [73]. If the sum score (range: 0–24) was nine or higher, indicating at least moderate psychological distress, they were invited to participate in the study. Additionally, we checked whether potential participants wished to obtain employment subject to social insurance contributions. If all criteria were met, participants were informed about the study’s procedure and content, including their eligibility based on their reported psychological distress, and written informed consent was obtained. Participation in the data collection was incentivized with compensation for time and travel costs.

We recruited 455 individuals for the study, with complete data regarding the main study variables (i.e., self-stigma, job-search self-efficacy, and job-search behavior) being provided by 422 individuals. The in the analysis included participants’ average age was 38.4 years (*SD* = 11.1), ranging from 19 to 60 years, and 58.1% were male. They had on average 0.9 children (*SD* = 1.3) and were predominantly single (55.2%). In terms of highest educational degree, most participants reported a lower secondary school leaving certificate (19.7%) or a vocational training certificate (i.e., a completed apprenticeship; 32.5%). A considerable number of participants reported having an upper secondary education with university entrance qualification (12.6%) or a university degree (16.8%). In line with the study inclusion criterion of a basic knowledge of German, the majority of participants was born in Germany (76.1%) and/or had German citizenship (79.3%). The median of participants’ monthly household net income was €819.00. The median of participants’ self-reported duration of current unemployment was 7.0 months. Participants’ psychological distress was on average 14.6 (*SD* = 3.8), ranging from 9 to 24, indicating that the average participant was at risk for severe mental illness (cut-off > 13; [74]). More than half of the participants reported having been diagnosed with at least one mental health condition in the past (56.2%), with diagnoses predominantly belonging to affective disorders, anxiety-related disorders and substance use disorders. A considerable number of those diagnosed reported more than one diagnosis (51.5%); with the most frequent comorbidity being depression and anxiety (19.4% of all those with diagnosis), depression and substance use disorders (14.8%), and depression and post-traumatic stress disorder (12.2%). The demographic variables are displayed in Table 1.

**Table 1:** Sociodemographic characteristics of the study sample

Variable	Types of descriptive statistics or categories	Descriptive statistics
Age (years)	M (SD) Range	38.4 (11.1) 19–60
Gender *N* (%)	Male	245 (58.1)
	Female	176 (41.7)
	Diverse	1 (0.2)
Marital status *N* (%)	Single	233 (55.2)
	In a relationship < 1 year	13 (3.1)
	In a relationship ≥ 1 year	47 (11.1)
	Married / Registered partnership	38 (9.0)
	Divorced	51 (12.1)
	Widowed	2 (0.5)
	Separated	38 (9.0)
Number of children *N (%)*	0	254 (60.2)
	1	61 (14.5)
	2	52 (12.3)
	3	36 (8.5)
	≥ 4	19 (4.4)
Education *N* (%)	No formal educational qualification	12 (2.8)
	Lower secondary school leaving certificate	83 (19.7)
	Upper secondary school leaving certificate	58 (13.7)
	Vocational training certificate	137 (32.5)
	Upper secondary education with university entrance qualification	53 (12.6)
	Vocational college	8 (1.9)
	University degree	71 (16.8)
Country of birth *N* (%)	Germany	321 (76.1)
	Other	101 (23.9)
Citizenship *N* (%)	German only	315 (74.6)
	Dual citizenship (i.e., German with other)	20 (4.7)
	Other	87 (20.6)
Disposable household income per month (in Euro)	*M* (*SD*) Median (IQR) Range	886.1 (678.2) 819.0 (620.0) 0.0–4,400.0
Duration of self-reported current unemployment (in months)	*M* (*SD*) Median (IQR) Range	17.9 (31.4) 7.0 (15.0) 0.0-315.0
Mental health diagnosis *N (%)*	Affective disorders (e.g., depressive disorder, bipolar disorder)	196 (46.4)
	Anxiety-related disorders (e.g., anxiety disorder, post-traumatic stress disorder, obsessive-compulsive disorder)	109 (25.8)
	Substance use disorders	49 (11.6)
	Schizophrenia spectrum and other psychotic disorders	16 (3.8)
	Personality disorders (e.g., borderline avoidant)	24 (5.7)
	Attention-deficit hyperactivity disorders	17 (4.0)
	Other mental health diagnoses	28 (6.6)

*Note. N* = 422

### Measures

Self-stigma, job-search self-efficacy, psychological distress, and social inclusion were measured with established scales as part of a questionnaire, whereas data on job-search behavior, readiness to find employment, and diagnosis of mental health conditions were collected as part of a structured interview.

#### Self-stigma

Self-stigma was measured with the apply-subscale of the *Self-Stigma of Mental Illness Scale – Short Form* [75]. It consisted of five items and responses were given on a 9-point Likert scale ranging from 1 (*strongly disagree*) to 9 (*strongly agree*). As some participants may not necessarily have a diagnosed mental health condition, the original introductory phrase was changed from “Because I have a mental illness, I …” to “Because I have mental health issues, I …”, for instance, “… will not recover or get better”. The self-stigma score was computed by summing the items. Cronbach’s alpha was 0.66.^1^

#### Job-search self-efficacy

Job-search self-efficacy was measured with the *Job-Search Self-Efficacy Scale* by Caplan et al. [37]. Participants responded to six items relating to job-search activities (e.g., “completing a good job application and resume”, “making the best impression in a job interview”) in terms of how confident they felt performing these activities on a scale from 1 (*not at all confident*) to 5 (*great deal confident*). Items were averaged to compute the score. Cronbach’s alpha was 0.87.

#### Job-search behavior

In the structured interview, participants were asked in which job-search activities they engaged in the last 12 months, besides registering with the job center and working with employment advisors there. The interviewer started with an open question regarding such activities. The interview schedule contained a list of 12 common job-search activities (e.g., submitting a job application in response to job advertisements, seeking help from a job coach, participating in a job application training), which the interviewer ticked off when mentioned by participants. Other reported relevant job-search activities (e.g., drawing on one’s social network to gain employment) were noted down by the interviewer. After the participants’ report on the open question, the interviewer went through the list of job-search activities, explicitly enquiring about the not yet mentioned activities. We calculated the sum of participants’ reported activities to represent the intensity of their job-search behavior (cf [57, 76]). The standardized interview procedure was used to enhance reliability across participants. Furthermore, interview experiences were discussed in the research group to align how activities were categorized, either into the provided categories or new categories.

#### Social inclusion

Social inclusion was measured with the *Experiences of Social Inclusion Scal*e by Leemann et al. [54]. The scale consists of 10 items (e.g., “I belong to a group or community that is important for me”, “I feel that my life has purpose”). Participants indicated their agreement with the items on a 5-point Likert scale ranging from 1 (*strongly disagree*) to 5 (*strongly agree*). The scale score was obtained by summing up the items. Cronbach’s alpha was 0.83.

#### Readiness to find employment

After conducting the structured interview, the interviewers rated the interviewee’s readiness to find employment by categorizing them into the five stages of behavioral change of the transtheoretical model of change: precontemplation, contemplation, preparation, action, and maintenance [60]. To rate these stages, interviewers were given behavioral descriptions of how this stage could manifest. Furthermore, we discussed the interviewers’ experiences with the participants to align the scoring of this variable. The marginal stages were, however, underrepresented in our sample: Only 2.6% of individuals were in the precontemplation stage, whereas 4.7% were in the maintenance stage.^2^ We decided to join the marginal stages with their adjoint stage (i.e., precontemplation and contemplation; action and maintenance). Accordingly, we had three groups of low (21.1%), moderate (39.1%), and high (39.8%) readiness to find employment.

#### Psychological distress

Psychological distress was measured with the Kessler’s Psychological Distress Scale – K6 version [73]. The scale consists of six items describing feelings of distress (e.g., “nervous”, “restless or fidgety”). Participants reported the frequency of experiencing these feelings within the last 30 days on a 5-point frequency scale ranging from 0 (*never*) to 4 (*all the time*). The scale score was calculated by summing up the items. Cronbach’s alpha was 0.68.

#### Diagnosis of mental health condition

As part of the structured interviews, participants were asked whether they had ever been diagnosed with a mental health condition by a medical or mental health professional. The participants’ responses were coded into a binary variable indicating the presence (= 2) or absence (= 1) of a diagnosed mental health condition. In addition, participants reported the specific diagnoses, which were categorized into overarching diagnostic groups. Diagnoses were either selected from a predefined list or recorded verbatim and subsequently categorized during data cleaning by trained psychologists and psychotherapists. This classification into diagnostic groupings was discussed in the research team to ensure consistent application of the coding scheme.

### Analysis strategy

#### Variable-centered approach

To test the Hypotheses 1 to 6, we conducted structural equation modeling with Mplus 8.11 [77], using robust maximum likelihood estimation to evaluate the direct effects. To examine the indirect effect of self-stigma on job-search behavior (Hypothesis 1; Step 1), we used bias-corrected bootstrap confidence intervals (CI; 1,000 bootstrap resamples). To test the interactions and conditional indirect effects (Hypotheses 3, 5–6), we first added the direct effect of the respective moderator variable onto job-search self-efficacy to the model (Step 2), then the interaction term between self-stigma and the moderator variable (Step 3). To assess the conditional indirect effects, we computed the index of moderated mediation following Hayes [78], using bias-corrected bootstrap CIs based on 1,000 bootstrap resamples. To test Hypothesis 4 regarding readiness to find employment, we conducted a multigroup comparison to assess whether the indirect effect between self-stigma and job-search behavior via job-search self-efficacy significantly differed in the three levels of readiness.

#### Person-centered approach

To identify subpopulations in the sample, we conducted latent profile analysis using five indicator variables [79]: Self-stigma, psychological distress, and social inclusion as continuous variables, retained in their original scale values, and readiness to find employment and diagnosis of a mental health condition as categorical variables. We started with a one-profile solution and successively increased the number of profiles until the model fit no longer improved [80]. We estimated the profiles with the manifest scale scores of the included variables, using Mplus 8.11 [77] with the maximum likelihood estimator with robust standard errors (MLR). All models were estimated with 3000 random sets of start values, 100 iterations, and 100 solutions retained for final optimization in order to avoid suboptimal local solutions [81, 82].

To select the most suitable profile solution, we drew on the following fit indices: the Bayesian information criterion (BIC), the sample-size adjusted BIC (SABIC), the adjusted Lo-Mendell-Rubin likelihood ratio test (aLRT), and the bootstrapped likelihood ratio test (BLRT) [80]. For the BIC and SABIC, a lower value indicates a better fitting model. The aLRT and BLRT compare a *k*-profile model with a *k-1*-profile model, with a significant difference indicating that the *k*-profile model is the better fitting model. Additionally, we plotted the BIC values of the profile solutions to examine when the decrease in BIC flattened out to assist identifying the optimal number of profiles (i.e., “elbow plots” [83, 84]). If an additional profile contained a low number and proportion of cases, we considered rejecting this additional profile [80]. We selected the final number of profiles based on the fit indices as well as the meaningful interpretability of the profile solution. We did not use the entropy values to determine the number of profiles, but rather to assess the classification accuracy, with higher values (ranging from 0.00 to 1.00) indicating a better accuracy [85].

Once the optimal number of profiles was selected, we used the Bolck-Croon-Hagenaars (BCH) command in Mplus to examine whether the profiles differ in terms of the profile members’ level of job-search self-efficacy. This command based on Bolck et al. [86] enables a three-step approach to obtain unbiased estimates of profile-specific means for outcome variables [77, 87].The significance of differences in job-search self-efficacy based on profile membership was assessed using Wald tests.

## Results

Table 2 presents descriptive statistics, intercorrelations, and internal consistencies of the study variables. Intercorrelations show that self-stigma is negatively associated with job-search self-efficacy and job-search behavior, while self-efficacy and behavior are positively related. Among the proposed boundary conditions, social inclusion and readiness to find employment (i.e., the proposed resources) are positively linked to job-search self-efficacy; psychological distress is negatively related, and a diagnosed mental health condition shows no association (i.e., the proposed stressors). The internal consistency of the self-stigma scale was slightly below the cut-off score of acceptable reliability (α = 0.66). In an original test of the short-form of the Self-Stigma of Mental Illness Scale, the internal consistency of the apply-subscale in a German sample of individuals with diagnosed mental health conditions was 0.74 [75]. The lower coefficient could partially be due to applying the scale to a sample of individuals with mental health issues with an accordingly adjusted scale instruction.

### Results from the variable-centered approach

#### Test of indirect effects

We proposed a negative direct effect of self-stigma on job-search self-efficacy (Hypothesis 1) as well as a negative indirect effect on job-search behavior via job-search self-efficacy (Hypothesis 2). Self-stigma was negatively associated with job-search self-efficacy (*b* = -0.27, *SE* = 0.07, *p* < .001), supporting Hypothesis 1. Job-search self-efficacy, in turn, was positively associated with job-search behavior (*b* = 0.14, *SE* = 0.05, *p* = .008), whereas self-stigma showed no *direct* effect on job-search behavior (*b* = -0.09, *SE* = 0.07, *p* = .17). Supporting Hypothesis 2, there was a significant negative *indirect* effect between self-stigma and job-search behavior via job-search self-efficacy (indirect effect = -0.037, *SE* = 0.016, 95% CI [-0.078; -0.012]).

**Table 2:** Means, standard deviations, intercorrelations, and internal consistencies of study variables

Variable	*M* (*SD*)	1	2	3	4	5	6	7
1. Self-stigma	15.01 (7.40)	0.66						
2. Job-search self-efficacy	3.39 (0.96)	− 0.25***	0.87					
3. Job-search behavior	1.60 (1.44)	− 0.11*	0.18***	-				
4. Social inclusion	35.27 (7.48)	− 0.36***	0.48***	0.10	0.83			
5. Readiness to find employment ^a^	2.19 (0.76)	− 0.19***	0.25***	0.41***	0.17***	-		
6. Psychological distress	14.58 (3.83)	0.29***	− 0.16**	− 0.07	− 0.29***	− 0.11*	0.68	
7. Diagnosis of mental health condition	1.56 (0.50)	0.15**	− 0.08	− 0.08	− 0.13*	− 0.09	0.12*	-

*Note. N* = 422. Cronbach’s alpha coefficients are reported along the diagonal. Diagnosis of mental health condition: 1 = no, 2 = yes

^a^ The correlations of readiness to find employment with the other study variables represent Spearman’s rho correlation coefficients

*p < .05. **p < .01. ***p < .001

**Table 3:** Results from structural equation modeling for the direct path estimates and estimates for the conditional indirect effects

Direct effects on job-search self-efficacy				
Variable	Mod: Social inclusion		Mod: Readiness to find employment	
	*b*	*SE*	*b*	*SE*
Step 2				
Self-stigma	-0.05	0.06	-0.22**	0.07
Moderator	0.54***	0.08	0.27***	0.07
Step 3				
Self-stigma	-0.05	0.06	n/a	n/a
Moderator	0.55***	0.08	n/a	n/a
Interaction	-0.03	0.04	n/a	n/a

Conditional indirect effects on job-search behavior						
	Estimate	*SE*	95% CI [LL; UL]	Estimate	*SE*	95% CI [LL; UL]
IMM	-0.003	0.007	[-0.019; 0.007]	n/a	n/a	n/a
At low mod	-0.004	0.012	[-0.035; 0.016]	-0.012	0.043	[-0.179; 0.022]
At mean mod	-0.007	0.010	[-0.033; 0.007]	-0.022	0.021	[-0.085; 0.002]
At high mod	-0.010	0.012	[-0.042; 0.006]	-0.004	0.026	[-0.060; 0.047]

Direct effects on job-search self-efficacy				
Variable	Mod: Psychological distress		Mod: Diagnosis of mental health condition ^a^	
	*b*	*SE*	*b*	*SE*
Step 2				
Self-stigma	-0.22**	0.08	-0.26***	0.08
Moderator	-0.12	0.08	-0.11	0.11
Step 3				
Self-stigma	-0.21**	0.07	-0.30*	0.12
Moderator	-0.12	0.08	-0.10	0.11
Interaction	-0.03	0.07	0.07	0.13

Conditional indirect effects on job-search behavior						
	Estimate	*SE*	95% CI [LL; UL]	Estimate	*SE*	95% CI [LL; UL]
IMM	-0.005	0.011	[-0.028; 0.016]	0.010	0.020	[-0.021; 0.060]
At low mod	-0.024	0.018	[-0.074; 0.001]	**-0.042**	0.022	[-0.106; -0.011]
At mean mod	**-0.029**	0.015	[-0.069; -0.006]			
At high mod	**-0.034**	0.019	[-0.085; -0.005]	**-0.032**	0.016	[-0.081; -0.008]

*Note. N* = 422. Unstandardized estimates reported. Indices of moderated mediation and indirect effect estimates were calculated using bias-corrected bootstrap confidence intervals (1,000 resamples). Significant estimates based on the 95% CI are marked in bold

*IMM *Index of moderated mediation,* CI C*onfidence interval,* LL *Lower limit,* UL *Upper limit

^a^ Diagnosis of mental health condition: Low level of moderator = not having a diagnosis; high level of moderator = having a diagnosis

*p < .05. **p < .01. ***p < .001

#### Test of conditional indirect effects

We proposed four moderators which were hypothesized to affect the indirect effect between self-stigma and job-search behavior via job-search self-efficacy. Table 3 displays the direct path estimates regarding job-search self-efficacy, the estimates for the indices of moderated mediation, and the indirect effects at different levels of the respective moderator.

##### Social inclusion

Hypothesis 3 proposed that social inclusion would weaken the negative indirect effect between self-stigma and job-search behavior. We neither found a significant interaction between self-stigma and social inclusion in terms of job-search self-efficacy (*b* = -0.03, *SE* = 0.04, *p* = .55), nor a significant index of moderated mediation (index = -0.003, SE = 0.007, 95% CI [-0.019; 0.007]), meaning that the level of social inclusion did not affect the indirect effect of self-stigma on job-search behavior via job-search self-efficacy. Hypothesis 3 therefore had to be rejected. Adding social inclusion to the model showed a positive main effect on job-search self-efficacy, indicating the relevance of feeling socially included on feeling confident when searching employment.

##### Readiness to find employment

With Hypothesis 4, we proposed that the readiness to find employment would mitigate the negative indirect effect between self-stigma and job-search behavior. The multi-group comparison showed no significant differences in the indirect effects in the three levels of readiness (diff_1vs2_ = 0.010, *SE* = 0.028, *p* = .71; diff_2vs3_ = -0.018, *SE* = 0.031, *p* = .56). Accordingly, the indirect effect of self-stigma on job-search behavior via job-search self-efficacy was not conditional on the level of readiness to find employment, thus Hypothesis 4 was not supported.

**Fig. 2:**
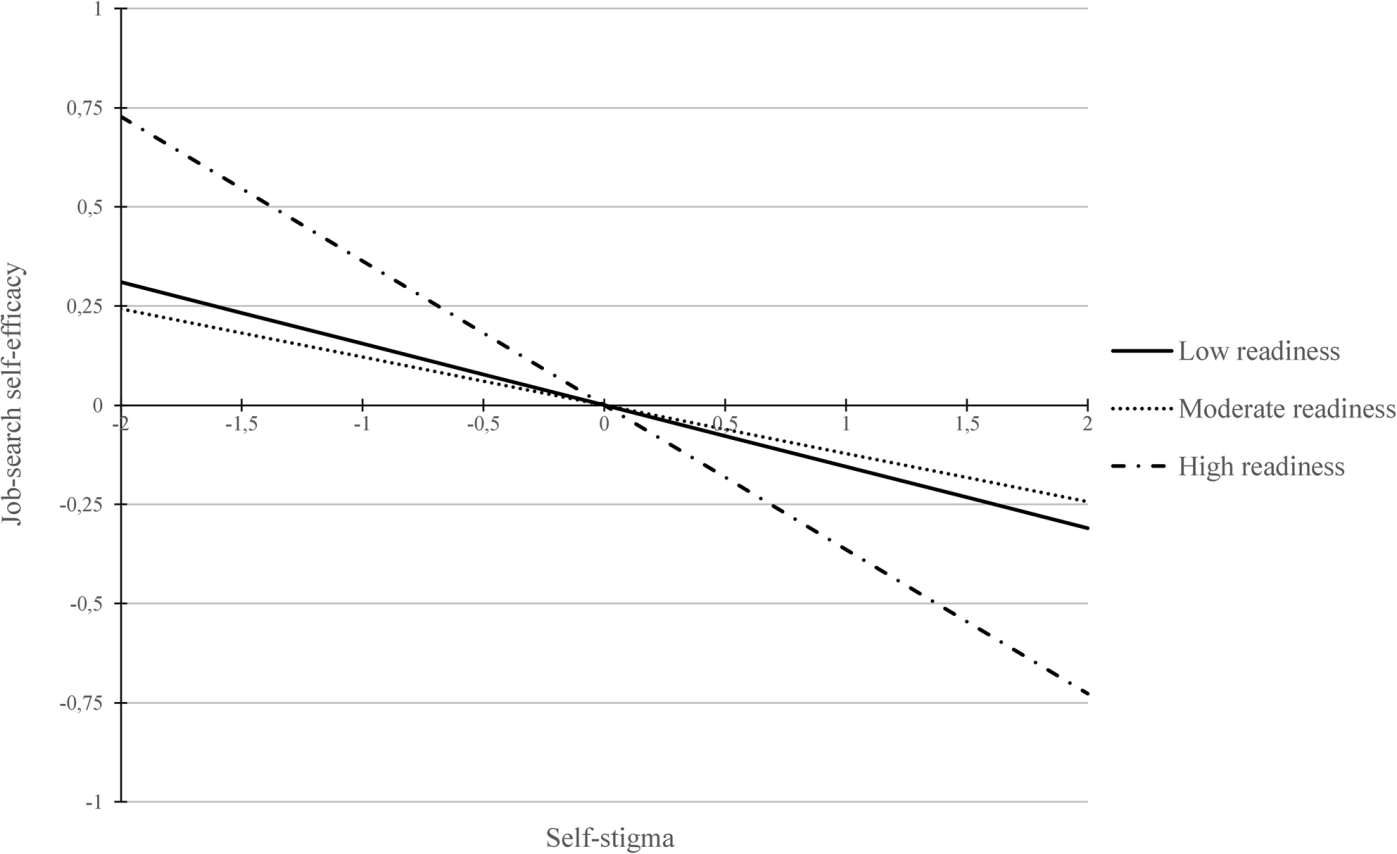
Interaction effect between self-stigma and readiness to find employment. Note: As latent factors, job-search self-efficacy and self-stigma are standardized

Although the indirect effect was not conditional on the level of readiness, we did find that the direct effect between self-stigma and job-search self-efficacy differed in the three levels: Whilst there was no significant direct effect in the group of low (*b* = -0.16; *SE* = 0.23, *p* = .50) and moderate readiness (*b* = -0.12; *SE* = 0.08, *p* = .11), there was a significant effect for high readiness (*b* = -0.36; *SE* = 0.10, *p* < .001). The direct effects in the first two levels did not significantly differ (diff_1vs2_ = 0.03, *SE* = 0.24, *p* = .89), but there was a significant difference between the direct effects of moderate and high readiness (diff_2vs3_ = -0.24, *SE* = 0.12; *p* = .04). Exploring this further, contrary to our hypothesis, the negative effect did not become weaker, but stronger with increasing readiness to find employment. Specifically, if participants were perceived to be highly ready in terms of finding employment, then self-stigma affected them *more* negatively in terms of their confidence in their job-search capabilities (see Fig. 2). This effect should be considered exploratory and interpreted cautiously.

##### Psychological distress

Hypothesis 5 proposed that psychological distress would strengthen the negative indirect effect between self-stigma and job-search behavior. We neither found a significant interaction between self-stigma and psychological distress in terms of job-search self-efficacy (*b* = -0.03, *SE* = 0.07, *p* = .62), nor a significant index of moderated mediation inclusion (index = 0.005, *SE* = 0.011, 95% CI [-0.028; 0.016]), indicating that the indirect effect of self-stigma on job-search behavior via job-search self-efficacy was not conditional on the level of psychological distress, thus not supporting Hypothesis 5.

##### Diagnosis of mental health condition

Hypothesis 6 posited that having a diagnosed mental health condition would exacerbate the negative indirect effect between self-stigma and job-search behavior. There was no significant interaction between self-stigma and having a diagnosis in terms of job-search self-efficacy (*b* = 0.07, *SE* = 0.13, *p* = .58) and no significant index of moderated mediation (index = 0.010, *SE* = 0.020, 95% CI [-0.021; 0.060]), indicating that having a diagnosed mental health condition did not affect the indirect effect of self-stigma on job-search behavior via job-search self-efficacy. Accordingly, Hypothesis 6 had to be rejected.^3^

### Results from the person-centered approach

#### Identification of profiles

**Fig. 3:**
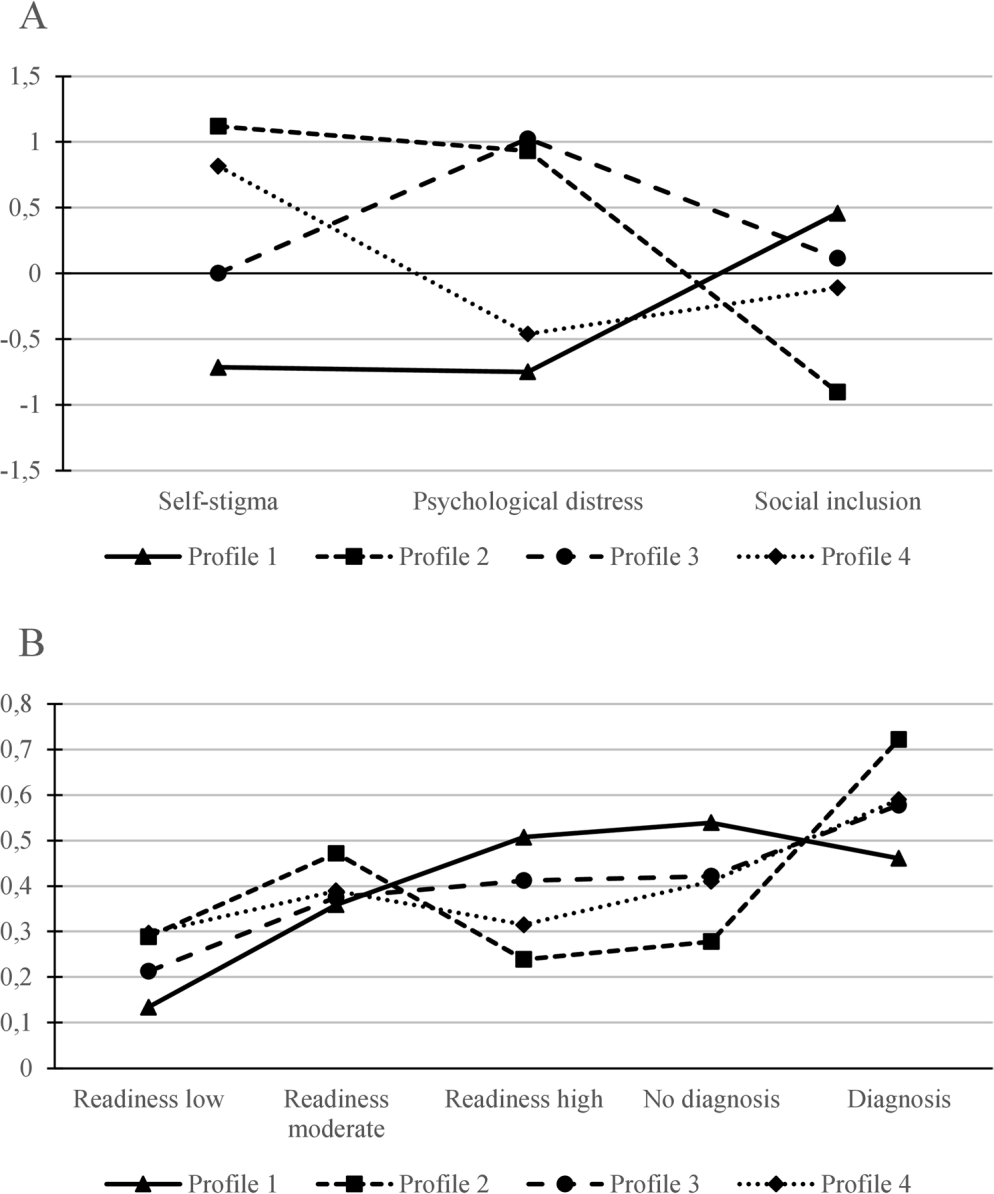
Final latent profile solution illustrating the means and conditional response probabilities. Note: **A**: Mean scores of continuous variables (i.e., self-stigma, social inclusion, psychological distress); standardized mean scores are displayed for better comparison. **B**: Conditional response probabilities (CRP) for categorical variables (i.e., readiness to find employment, diagnosis)

The fit indices of the estimated models are reported in Table 4. The biggest reduction in the BIC was between the 1- and 2-profile solutions. In the subsequent profile solutions, the BIC increased slightly. The SABIC, on the other hand, decreased up to the 5-profile solution, again with the biggest drop between the 1- and 2-profile solutions. The aLRT indicated a significant difference between the 1- and 2-profile solutions, whereas the BLRT indicated significant differences between all solutions up until the 5-profile solution. One of the profiles in the 5-profile solution included only 9.5% of cases. Balancing the fit indices, profile sizes, and interpretability, we selected the 4-profile solution. This solution is illustrated in Fig. 3: Panel A graphs the standardized means of the continuous variables, Panel B displays the conditional response probabilities per category of the categorical variables.

**Table 4:** Fit statistics from the latent profile analysis

No. of profiles	MLL	#FP	BIC	SABIC	aLRT (*p*)	BLRT (*p*)	Entropy
1 Profile	-4736.718	9	9527.841	9499.281	n/a	n/a	n/a
2 Profiles	-4655.481	16	9407.682	9356.909	< 0.001	< 0.001	0.693
3 Profiles	-4639.290	23	9417.615	9344.629	0.279	< 0.001	0.719
4 Profiles	-4617.525	30	9416.400	9321.201	0.071	< 0.001	0.718
5 Profiles	-4605.009	37	9433.683	9316.269	0.321	0.008	0.746
6 Profiles	-4595.906	44	9457.793	9318.167	0.756	0.158	0.774

*MLL* Model log likelihood, *#FP* number of free parameters, *BIC* Bayesian information criteria, *SABIC* Sample-size adjusted BIC, *aLMR* adjusted Lo-Mendel-Rubin likelihood ratio test, *BLRT* Bootstrapped likelihood ratio test

Profile 1, the largest profile (41.38%), is characterized by low levels of self-stigma (*M* = 9.72) and psychological distress (*M* = 11.71), and moderate to high levels of social inclusion (*M* = 38.68). In this profile, the categories of high readiness to find employment (CPR = 0.51) and not having a mental health diagnosis (CPR = 0.54) have the highest conditional response probabilities (CPR).

Profile 2 contains 20.53% of cases of participants with high levels of self-stigma (*M* = 23.27) and psychological distress (*M* = 18.15), and low levels of social inclusion (*M* = 28.52). Participants in Profile 2 are more likely to be in the category of moderate readiness to find employment (CPR = 0.47) and to have a diagnosis of a mental health condition (CPR = 0.72).

Profile 3, containing 19.86% of cases, can be described by low levels of self-stigma (*M* = 11.95), high levels of psychological distress (*M* = 18.50), and moderate levels of social inclusion (*M* = 36.12). In this profile, participants are similarly likely to be in the category of high readiness to find employment (CPR = 0.41) or moderate readiness (CPR = 0.38), and more likely to be in the category of having a mental health diagnosis (CPR = 0.58).

Lastly, Profile 4, containing 18.23% of cases, is characterized by participants with high levels of self-stigma (*M* = 21.04), lower levels of psychological distress (*M* = 12.82), and moderate levels of social inclusion (*M* = 34.44). Furthermore, participants in Profile 4 are more likely to be in the category of moderate readiness to find employment (CPR = 0.39) and having a mental health diagnosis (CPR = 0.59).

Contrasting the profiles, for self-stigma, we found that Profiles 2 and 4 had the highest levels, Profile 1 the lowest and Profile 3 in between. In terms of psychological distress, Profiles 2 and 3 had higher levels than Profiles 4 and 1, while Profiles 2 and 3 did not differ significantly; Profile 4 was slightly higher than Profile 1. For social inclusion, we found the highest level in Profiles 1 and 3, with them not differing significantly, then Profile 4, and Profile 2 having the lowest level. Profiles 1 and 2 differed in terms of readiness to find employment. Specifically, members of Profile 1 had higher odds of moderate and high readiness relative to Profile 2, indicating higher overall motivation in Profile 1. Furthermore, members of Profile 1 were also more likely to be rated moderately or highly ready to find employment than those in Profile 4. No other significant differences in readiness levels were observed. Regarding mental health diagnosis, Profiles 1 and 2 differed such that members of Profile 2 had a higher probability of reporting a diagnosed mental health condition compared to those in Profile 1. No other profile comparisons yielded significant differences in diagnosis status. The statistical test values for these comparisons are listed in Table 5 for the continuous variables and in Table 6 for the categorical variables.

**Table 5:** Profile comparisons based on continuous indicator variables

Profile	*M*	*SE*	Comparison to Profile 1 Wald *|z|*	Comparison to Profile 2 Wald *|z|*	Comparison to Profile 3 Wald *|z|*
**Self-stigma**					
Profile 1	9.72	0.68			
Profile 2	23.27	1.44	7.07***		
Profile 3	11.95	0.66	2.29*	8.43***	
Profile 4	21.04	1.55	9.57***	0.86	5.48***
**Psychological distress**					
Profile 1	11.71	0.27			
Profile 2	18.15	0.41	-12.24***		
Profile 3	18.50	0.49	15.15***	0.50	
Profile 4	12.82	0.45	2.04*	-11.69***	8.99***
**Social inclusion**					
Profile 1	38.68	0.58			
Profile 2	28.52	1.06	8.39***		
Profile 3	36.12	1.53	1.48	3.49***	
Profile 4	34.44	0.88	3.98***	4.51***	-0.91

Note. Each pairwise comparison was tested with degrees of freedom df = 1

**Table 6:** Profile comparisons based on categorical indicator variables

**Conditional response probabilities**						
	Readiness				Diagnosis	
Profile	low	moderate		high	no	yes
Profile 1	0.13	0.36		0.51	0.54	0.46
Profile 2	0.29	0.47		0.24	0.28	0.72
Profile 3	0.21	0.38		0.41	0.42	0.58
Profile 4	0.30	0.39		0.32	0.41	0.59

**Contrasting profiles – Odds ratios [CI]**						
	Readiness > low		Readiness > moderate		Diagnosis – yes vs. no	
Profile 1 vs. 2	**2.62 [1.25; 5.49]**		**3.26 [1.68; 6.35]**		**0.33 [0.17; 0.63]**	
Profile 3 vs. 1	0.57 [0.24; 1.40]		0.68 [0.32; 1.44]		1.60 [0.82; 3.13]	
Profile 4 vs. 1	**0.37 [0.16; 0.86]**		**0.45 [0.21; 0.97]**		1.68 [0.74; 3.82]	
Profile 3 vs. 2	1.50 [0.55; 4.12]		2.23 [0.87; 5.73]		0.53 [0.23; 1.19]	
Profile 4 vs. 2	0.97 [0.39; 2.38]		1.46 [0.58; 3.68]		0.55 [0.22; 1.42]	
Profile 4 vs. 3	0.64 [0.24; 1.69]		0.66 [0.25; 1.72]		1.05 [0.45; 2.43]	

Note. Odds ratios > 1 indicate higher odds in the first-mentioned profile. Profile numbering differed between the Mplus output and the manuscript, resulting in different comparative directions. Significant estimates based on the 95% confidence interval are marked in bold

CI confidence interval

In conclusion, regarding Research Question 1, we identified four distinct profiles of unemployed individuals with mental health issues that differed both quantitatively (i.e., in the level of the indicators) and qualitatively (i.e., in the configurations of indicator patterns) [82].

#### Outcomes of profile membership

Regarding Research Question 2, we examined how the identified profiles differed regarding job-search self-efficacy. The overall Wald chi-square test indicated significant differences in job-search self-efficacy across the four profiles (Wald χ² (3) = 47.42, *p* < .001): Job-search self-efficacy was the lowest in Profile 2. In Profile 4, job-search self-efficacy was significantly higher than in Profile 2, but significantly lower than in Profile 1. Profile 4 and 3 did not differ significantly in terms of job-search self-efficacy, neither did Profiles 1 and 3. Consequently, there are some quantitative differences regarding job-search self-efficacy. Table 7 displays the scores for the pairwise difference tests.

**Table 7:** Three-step results for distal outcomes of profile membership

Profile			Comparison to Profile 1 Wald χ^2^	Comparison to Profile 2 Wald χ^2^	Comparison to Profile 3 Wald χ^2^
	*M*	*SE*			
Profile 1	3.72	0.08			
Profile 2	2.81	0.12	42.60***		
Profile 3	3.51	0.15	1.35	11.81***	
Profile 4	3.19	0.13	10.85***	4.11*	2.58

Note. Each pairwise comparison was tested with degrees of freedom df = 1

*p < .05. **p < .01. ***p < .001

## Discussion

The present study used two complementary approaches to examine why unemployed individuals with mental health issues struggle to find employment due to low confidence in their job-search abilities and low activation in their job search. A particular focus was on the role of self-stigma which had been proposed as impediment to finding re-employment [19], but has been empirically under-research. In terms of the variable-centered approach, we found that self-stigma negatively affected job-search behavior by lowering job-search self-efficacy. The proposed boundary conditions did not moderate this indirect effect. Participants’ readiness to find employment moderated the direct association between self-stigma and job-search self-efficacy, such that the association was stronger at higher levels of readiness. In terms of the person-centered approach, we identified four distinct profiles which differed both in their configuration and their association with job-search self-efficacy.

### Implications for theory and research

Our variable-centered analysis supports a domain-specific “why try” effect in the context of unemployment [27]: Unemployed individuals with mental health issues and elevated self-stigma report lower job-search self-efficacy, consequently, reduced job-search behavior. Although job-search behavior constitutes a problem-focused coping strategy to obtain employment, individuals might avoid this effortful strategy due to anticipated failure. Extending prior research on the “why try” effect, our findings indicate that this effect is not limited to those with formal mental health diagnoses but can also occur in undiagnosed individuals with mental health issues. While self-stigma was slightly more pronounced among individuals with a diagnosis, the indirect effect was not conditional on diagnostic status, suggesting that self-labeling processes may already be active in this sample.

We found no empirical support for conditional indirect effects in the self-stigma–job-search behavior pathway via job-search self-efficacy. The non-significant findings may be attributable to characteristics of the sample and the methodological approach. The sample primarily comprised individuals with common mental health conditions or no formal diagnosis, which may have resulted in restricted variability in self-stigma, limiting the detection of interaction effects with the proposed moderators. In addition, although the sample size was considerable, the small effect sizes observed for the associations between self-stigma, job-search self-efficacy, and job-search behavior—combined with the complexity of the tested model—may have resulted in insufficient statistical power. Finally, the proposed moderators may have been too generic for this population, suggesting that future research should employ more domain-specific moderators tailored to mental health–related and unemployment-related processes.

We did find an interactive effect between self-stigma and readiness to find employment on job-search self-efficacy, but it was not in line with our predictions: We considered readiness to find employment to be a resource for the job-search process, expecting a mitigating effect. In contrast, our analysis indicated that individuals who are on a higher stage of readiness show a stronger negative association between self-stigma and job-search self-efficacy. This unexpected moderating effect can be interpreted through the lens of the *“what matters most” framework* which posits that stigma affects individuals the most in domains which are highly important to them [88]. The framework emphasizes that stigma is especially harmful when it threatens access to culturally valued roles and identities [88]. In many societies, including Germany, employment is highly valued [89, 90], while unemployment is stigmatized [11, 15]. Accordingly, for individuals who are highly motivated and ready to re-enter employment, self-stigma due to mental illness may be especially detrimental because it directly undermines confidence in attaining a highly valued goal. In this context, self-stigma may intensify self-doubt and perceived inadequacy precisely among those who are most invested in succeeding in the job-search process. Consistent with this interpretation, Vinokur et al. [57] observed a trend that highly motivated unemployed individuals experience the greatest mental health decline due to unsuccessful job search. Furthermore, in this group of motivated but unsuccessful job-searchers, social support appeared to be an important buffer. We conclude from these findings that motivation and readiness for change—although being important drivers for job-search process—might put too much pressure on individuals who experience self-stigma. These individuals might benefit from strong social support that helps them to maintain their motivation and mental health throughout their frustrating job search.

With our person-centered analysis, we identified four distinct profiles which differed quantitatively and qualitatively regarding self-stigma, social inclusion, readiness to find employment, psychological distress, and having a mental health diagnosis. Profiles 1 and 2 showed mostly quantitative differences: Profile 1 represents the subgroup with the most favorable indicators: low levels of self-stigma and psychological distress, a lower likelihood of having a diagnosis, high perceived social inclusion, and a high readiness to find employment. Profile 2, on the other hand, reflects the most unfavorable profile configuration, characterized by high levels of self-stigma and psychological distress, the highest probability to have a diagnosis, low levels of social inclusion, and the lowest level of readiness. In contrast, the other two profiles differed qualitatively to each other and the other profiles in that they showed different configurations, particularly regarding psychological distress and self-stigma: Whereas individuals in Profile 3 indicated lower levels of self-stigma and higher levels of psychological distress, there was a reversed pattern in Profile 4. These profiles did not differ substantially regarding the other included variables. In terms of how the profile membership relates to job-search self-efficacy, Profile 1, the favorable profile, was associated with higher levels of job-search self-efficacy, whereas Profile 2, the unfavorable profile, with the lowest level. The two more qualitatively varied profiles were in between and did not differ significantly from each other in their job-search self-efficacy. However, whereas the level of job-search self-efficacy in Profile 4—with higher levels of self-stigma—differed significantly from Profile 1, Profile 3–with lower levels of self-stigma—did not. This indicates that profiles with high levels of self-stigma (i.e., Profiles 2 and 4) fared the worst in terms of job-search self-efficacy, despite varying in psychological distress and experiencing moderate social inclusion.

Applying the two complementary approaches allowed us to examine the process of how self-stigma might affect job-search behavior at the level of the population (i.e., via the variable-centered approach), as well as how various variables might interact and relate to job-search self-efficacy (i.e., via the person-centered approach). Both approaches indicated that self-stigma is negatively associated with job-search self-efficacy, emphasizing its relevance in the context of unemployment and mental health issues. The impeding role of high readiness to find employment, in contrast, did not become as apparent in the person-centered approach as in the variable-centered approach. Although the effects were rather small, our findings add to our understanding of the complex interplay of factors explaining why unemployed individuals with mental health issues struggle to find and maintain employment, warranting further research attention.

### Limitations and future research directions

Our study has a few limitations. The data is cross-sectional, therefore no clear conclusions regarding the directionality of the associations can be drawn. Future research should examine longitudinal data to examine the directionality further. Longitudinal data could also enable the examination of profile membership consistency across time and how membership changes might occur.

Furthermore, we measured job-search behavior retrospectively by asking participants about the range of their conducted job-search activities in the previous 12 months to approximate participants’ job-search intensity. We did not measure the frequency or number of individual activities (e.g., number of job applications), nor weight the activities based on how goal-oriented they are for a successful job search (e.g., submitting a job application vs. looking at job adverts). Blau [91], for instance, proposed to differentiate job-search behaviors into preparatory (e.g., revising application materials) and active behaviors (e.g., attending job interviews). Future research could apply job-search scales that distinguish between different types of activities to extend our understanding of how self-stigma affects job-search behavior.

In addition, job-search activities and mental health conditions were coded by interviewers based on participants’ self-reports using a standardized checklist, which introduces the possibility of misclassification or interviewer error. To mitigate potential inconsistencies, interview experiences and ambiguities in classifying reported activities and diagnoses were discussed within the research team during the early phase of data collection. Similarly, readiness to find employment was evaluated by the interviewers at the end of the interview based on the impression of the participants’ motivation to find employment, which may be subject to interviewer bias or misclassification. To enhance consistency, readiness stages were based on an established model of behavioral change [60], interviewers were provided with behavioral descriptions of each stage, and classification decisions were discussed within the research team. Future research should employ validated multi-item measures to more comprehensively assess readiness for change.

Furthermore, the interview data did not allow us to clearly determine the duration of participants’ job search at the time of the interview. When asked about the length of their unemployment, some respondents referred to the period of both being out of paid work and actively seeking employment (i.e., aligning with the formal definition of unemployment; cf [92]., while others referred only to the absence of paid employment, without seeking it (e.g., due to sick leave or caregiving responsibilities). Consequently, we could not ascertain whether participants had been actively job searching the preceding 12 months or had only recently initiated their search. This may have deflated the reported levels of job-search intensity, independent of self-stigma or job-search self-efficacy.

Another limitation is that self-stigma was assessed in individuals with mental health issues but not necessarily formally diagnosed mental health conditions, whereas the original scale was developed for diagnosed individuals. Therefore, the adapted scale may not have been fully applicable, potentially contributing to the scale’s lower internal consistency. The internal consistency observed in the present study may have led to bias in the effect estimates. Moreover, prior research suggests that self-stigma tends to be more pronounced among individuals with severe mental health conditions compared with those with common mental conditions [25, 93]. In this context, the present sample—characterized largely by common mental health conditions or the absence of a formal diagnosis—may have been less prone to self-stigma. Although the variability of self-stigma observed in the present study is comparable to that reported in other studies with more clinical samples [94], adapting and applying the scale to a population with relatively few individuals with severe mental health conditions may nevertheless have constrained the level and variability, especially in terms of the upper range of self-stigma. Future research could address this by recruiting larger and more diagnostically heterogeneous samples, thereby increasing variability in self-stigma.

Finally, the generalizability of our findings is limited given that the present sample was a rather specific sample of unemployed individuals with mental health issues who had recently started receiving basic income support in Germany. Study participation required sufficient knowledge of German, which may have excluded a notable portion of basic income support recipients in Germany. Future research should test the proposed research model and the replication of the identified profiles in a broader sample. It would be of particular interest to test the research model and profiles in other cultures, within Germany, but also globally, where mental health issues might be more or less stigmatized as this could affect levels of self-stigma as well as employability perceptions.

Future research should further examine moderators of the association between self-stigma and job-search self-efficacy. Drawing on COR theory, moderators that are conceptually closer to the lived context of unemployed individuals with mental health issues may be more informative. With regard to resources, future studies could move beyond broad indicators such as general social inclusion and instead investigate whether social support from the immediate social environment buffers the negative impact of self-stigma on job-search self-efficacy [25, 58]. Additional resources may include proactivity and coping style, as individuals who engage in more proactive and problem-focused coping may be better able to sustain job-search efforts in the presence of self-stigmatizing beliefs [58, 95]. On the other hand, further stressors may exacerbate the detrimental association between self-stigma and job-search self-efficacy. Longer and repeatedly unsuccessful job-search efforts may deplete personal resources through ongoing frustration and failure [11], thereby undermining emotional and motivational self-regulation and amplifying self-doubts associated with self-stigma. Finally, both anticipated and experienced workplace discrimination related to mental health issues may further exacerbate the negative impact of self-stigma on job-search self-efficacy, reinforcing expectations of rejection and reduced perceived chances of success [19, 96].

### Practical implications

Our findings have practical implications for interventions or training programs in the context of vocational rehabilitation, specifically addressing individuals with mental health issues. These implications focus predominantly on the role of self-stigma and how it could be alleviated at different levels. Firstly, self-stigma is internalized public stigma. Addressing public stigma is therefore an important leverage point for preventing and reducing self-stigma in individuals with mental health issues. Interventions which include social contact with individuals with lived experience, frequently referred to as peers, have been shown to be particularly effective for this purpose [16, 34].

Secondly, interventions can directly address individuals who experience self-stigma [16, 97]. One possible approach is the group-based, peer-led intervention *Honest*,* Open*,* Proud program* which offers guidance on coping with stigma and making disclosure decisions in various contexts. This intervention is promising in terms of stigma-stress and self-stigma [98]. Other existing interventions focus more explicitly on the cognitive component of self-stigma, aiming to effectively address these maladaptive beliefs with cognitive therapy approaches. For instance, *Narrative Enhancement and Cognitive Therapy* integrates cognitive restructuring with narrative work to help individuals challenge public stigma and self-stigma beliefs and develop more empowering self-concepts [97, 99].

Finally, self-stigma negatively affects self-worth, self-efficacy and willingness to keep trying to find employment. Being employed, on the other hand, can be associated with lower self-stigma [100], provided no discrimination is experienced at the workplace [101]. Thus, interventions should be offered which help individuals finding employment and supporting their mental health and self-efficacy during this frequently frustrating process [46, 102, 103]. In terms of finding employment, the *Individual Placement and Support* approach has been found to be effective for unemployed individuals with mental health conditions [104, 105]. Taken together, interventions offered to unemployed individuals with mental health issues and self-stigma should address different levels by integrating multiple components. Components, which have been shown to be effective in supporting unemployed individuals and/or individuals who self-stigmatize, could include enlisting social support—particularly from peers—to enable encouragement and vicarious learning, providing cognitive-therapeutic elements improving mental health, and job-search skills which boost self-efficacy through mastery [46, 102, 106].

## Conclusions

This study advances our knowledge of why unemployed individuals with mental health issues feel unconfident in their job-search capabilities and engage less in job-search behaviors, thus struggling to find re-employment. The variable-centered approach allowed us to examine the indirect effects between self-stigma and job-search behavior through job-search self-efficacy, supporting a domain-specific “why try” effect of self-stigma. With the person-centered approach, we found initial support for distinct profiles in a sample of unemployed individuals with mental health issues, indicating different configurations of variables in this context which affect job-search self-efficacy, thus highlighting the relevance of such approaches in this research context. Both approaches emphasize the relevance of self-stigma toward mental health issues in the job-search process, providing a leverage point for vocational rehabilitation.

## Data Availability

The data are not publicly available as participants did not explicitly consent to their data being shared with researchers outside the research team. Data is only available from the corresponding author on reasonable request after the publication of the final results of the wider intervention project.

## Footnotes

1 There was a mean difference in self-stigma depending on the status of a diagnosed mental health condition (*t*(420) = -3.15, *p* = .002, *d* = 0.31): Self-stigma was higher in the group with a diagnosed mental health condition (*M* = 16.00, *SD* = 7.41) than in the group without diagnosis (*M* = 13.74, *SD* = 7.21). The effect was small to moderate. Cronbach’s alpha was 0.65 for those individuals with diagnosis and 0.67 for those without.

2 This underrepresentation is unsurprising given the target group for the wider intervention study: Individuals in the precontemplation stage might be less likely to engage in a study aiming to support re-employment. In terms of maintenance, individuals in this stage would be more likely to already be in employment or far enough along in the process of finding re-employment that they would not consider themselves in need of such an intervention.

3 We probed the interaction effects for all proposed moderators between self-stigma and job-search self-efficacy. Other than readiness to find employment, the proposed moderating effects did not approach significance and were therefore not further explored.

## Abbreviations

aLMR: adjusted Lo-Mendel-Rubin likelihood ratio test
BIC: Bayesian information criteria
BLRT: Bootstrapped likelihood ratio test
CI: Confidence interval
COR: Conservation of resources
CRP: Conditional response probabilities
FP: number of free parameters
IMM: Index of moderated mediation
LL: lower limit of confidence interval
MLL: Model log likelihood
SABIC: Sample-size adjusted BIC
UL: Upper limit of confidence interval
